# Artificial intelligence-aided CT segmentation for body composition analysis: a validation study

**DOI:** 10.1186/s41747-021-00210-8

**Published:** 2021-03-11

**Authors:** Pablo Borrelli, Reza Kaboteh, Olof Enqvist, Johannes Ulén, Elin Trägårdh, Henrik Kjölhede, Lars Edenbrandt

**Affiliations:** 1 Region Västra Götaland, Department of Clinical Physiology, Sahlgrenska University Hospital, Gothenburg, Sweden; 2 Department of Electrical Engineering, Chalmers University of Technology, Gothenburg, Sweden; 3 Eigenvision AB, Malmö, Sweden; 4 Department of Clinical Physiology and Nuclear Medicine, Lund University and Skåne University Hospital, Malmö, Sweden; 5 Region Västra Götaland, Department of Urology, Sahlgrenska University Hospital, Gothenburg, Sweden; 6 Department of Urology, Institute of Clinical Science, Sahlgrenska Academy, University of Gothenburg, Gothenburg, Sweden; 7 Department of Molecular and Clinical Medicine, Institute of Medicine, Sahlgrenska Academy, University of Gothenburg, Gothenburg, Sweden

**Keywords:** Body composition, Muscles, Neural networks (computer), Subcutaneous fat, Tomography (x-ray, computed)

## Abstract

**Background:**

Body composition is associated with survival outcome in oncological patients, but it is not routinely calculated. Manual segmentation of subcutaneous adipose tissue (SAT) and muscle is time-consuming and therefore limited to a single CT slice. Our goal was to develop an artificial-intelligence (AI)-based method for automated quantification of three-dimensional SAT and muscle volumes from CT images.

**Methods:**

Ethical approvals from Gothenburg and Lund Universities were obtained. Convolutional neural networks were trained to segment SAT and muscle using manual segmentations on CT images from a training group of 50 patients. The method was applied to a separate test group of 74 cancer patients, who had two CT studies each with a median interval between the studies of 3 days. Manual segmentations in a single CT slice were used for comparison. The accuracy was measured as overlap between the automated and manual segmentations.

**Results:**

The accuracy of the AI method was 0.96 for SAT and 0.94 for muscle. The average differences in volumes were significantly lower than the corresponding differences in areas in a single CT slice: 1.8% *versus* 5.0% (*p* < 0.001) for SAT and 1.9% *versus* 3.9% (*p* < 0.001) for muscle. The 95% confidence intervals for predicted volumes in an individual subject from the corresponding single CT slice areas were in the order of ± 20%.

**Conclusions:**

The AI-based tool for quantification of SAT and muscle volumes showed high accuracy and reproducibility and provided a body composition analysis that is more relevant than manual analysis of a single CT slice.

## Key points

- Body composition measurements can give relevant prognostic information in specific clinical sttings.
- There is a need for reproductible and fast methods for body composition analysis.
- Computed tomography three-dimensional volumes proved to be more reliable than single-slice two-dimensional areas.
- Artificial intelligence-based tools are reliable and fast for body composition analysis.

## Background

Body composition has been shown to be associated with survival outcome in several of studies, both in oncological [1–6] and non-oncological patient groups [7]. One of the main applications of body composition analysis is to aid the diagnosis and management of sarcopenia. Sarcopenia usually is characterised as a progressive and generalised skeletal muscle loss and/or reduction of muscle function in elderly people and is associated with increased risk for falls, fractures, physical disability and mortality [8, 9]. In oncology, sarcopenia has been strongly associated with poor prognosis in a wide variety of malignancies [10].

Computed tomography (CT) and magnetic resonance imaging are usually considered as the best suitable methods for body composition analysis [9]. One of the most common approach to assess body composition is to measure the volumes of subcutaneous adipose tissue (SAT) and muscle on CT images. As part of the clinical workup for oncological patients, a CT examination is almost always present and available, either stand alone or as part of a positron emission tomography/CT (PET/CT) study.

Despite its prognostic value and the availability of CT in clinical practice, body composition is not routinely calculated. This is partly due to the fact that automated quantification tools are still under investigation and not widely available for clinical use. Another reason is the laborious work involved to manually segment SAT and muscle on CT images. The time burden for manual segmentations is probably the reason why, in many studies, SAT and muscle is only segmented on a single CT slice, *i.e*, using a two-dimensional (2D) approach, instead of a volumetric, three-dimensional (3D) assessment. The approximation of measurements obtained from a single CT slice (*i.e*, 2D measurements) to 3D fat and muscle volume measurements has shown to be poor [11]. Thus, there is an unmet need for an automated method for calculation of 3D volume of SAT and muscle in CT.

In the field of artificial intelligence (AI), deep learning methods offer new possibilities for automated analysis of medical images. Recently AI-based methods for automated analysis of body composition on CT images have been presented [12–16]. These methods are, however, trained to segment muscle and fat on single CT slices and not using the whole 3D volume. Our goal was to develop an AI-based method for automated quantification of 3D SAT and muscle volume from CT and to evaluate its accuracy and reproducibility in a separate test group of patients with prostate cancer.

## Methods

### Patients

The study was approved by the local research ethics at Universities of Gothenburg, Sweden (295-08;2016/103) and Lund, Sweden (LU552/2007). The AI-based method was trained using a retrospective set of CT scans from 50 patients with lymphoma, who had undergone ^18^F-fluorodeoxyglucose PET/CT examinations between January 2011 and August 2012. The patients of the training group (18 female and 32 male patients) had a mean age of 61 years (range 41–81) and a mean body weight of 78 kg (range 53–114).

A completely separate test set consisted of patients who had been part of a previous study investigating the value of PET/CT for staging of prostate cancer [17]. Each patient with biopsy-proven prostate cancer had undergone two PET/CT studies prior to treatment, one ^18^F-fluorocholine PET/CT and one ^18^F-fluoride PET/CT. From this retrospective group, we excluded five patients with more than two-week interval between the PET/CT studies, assuming that the volumes of SAT and muscle are relatively unchanged during a 2-week period. We also excluded five patients with hip prosthesis and corresponding metal artefacts in the CT images and five patients with limited field of view in one of the CT study. The remaining test group of 74 patients had a mean age of 67 years (range 50–76) and a mean body weight of 86 kg (range 54–120).

### Imaging

Training CT scans were acquired using an integrated PET/CT system (Biograph Truepoint 64; Siemens Healthineers, Erlangen, Germany). A low-dose CT scan (64-slice helical, 120 kVp, 30 mAs, 512 × 512 matrix) was obtained from the base of the skull to the mid-thigh, with a slice thickness was 3 mm. The test CT scans were acquired using an integrated PET/CT system (Philips Gemini TF, Philips Healthcare, Best, The Netherlands). A low-dose CT scan (16-slice helical, 120 kV, 50–300 mAs based on the patient’s total body mass, 512 × 512 matrix) was obtained from the base of the skull to the mid-thigh, with a slice thickness was 5 mm. The test set included CT scans obtained both with and without intravenous and/or oral contrast agents. The training and separate test studies were obtained from two different hospitals, Sahlgrenska University Hospital, Gothenburg, Sweden and Skåne University Hospital, Malmö/Lund, Sweden respectively. The PET images were not used in this study.

### Manual annotations

A cloud-based annotation tool (RECOMIA, www.recomia.org) was used for the annotation tasks in the training and test groups [18]. In the training studies, SAT and muscle were segmented by a nuclear medicine specialist experienced in CT annotations. Only SAT and muscle outside the peritoneal cavity were segmented; visceral adipose tissue (VAT) was not included. All CT slices from the cranial part of vertebrae T11 to the caudal part of the hip bone were segmented, resulting in 3D volumes of SAT and muscle. Density value thresholds were used during the segmentation process so that only voxels with HU between -190 and -30 were marked as SAT and voxels with HU between -30 and 150 were marked as muscle according to literature consensus for CT segmentation [6, 19, 20].

In the test set, two experienced nuclear medicine specialists with over 6 years of experience in reading PET/CT studies performed manual segmentations of SAT and muscle on a single CT slice at L3 vertebral level mid-point, as used in recently published studies [20–22]. The manual annotation task comprised of 50 3D segmentations in the training group and 148 2D segmentations in the test group for both SAT and muscle. The same definition of SAT and muscle and the same HU thresholds were applied as in the training set. The two studies from the same patients were segmented at different occasions with at least 3 days of time interval; in 35/74 patients (47%), studies were not segmented by the same physician. The areas of SAT and muscle were calculated from the manual 2D segmentations.

The quality of the AI-based segmentations in the test set was checked by one of the physicians. If a larger part of the body than from the cranial part of vertebrae T11 to the caudal part of the hip bone were included, *i.e*, the automated segmentation of vertebrae T11 and hip bone had failed, the SAT and muscle volumes of the slices outside the targeted body part were excluded. No other corrections of the automated segmentations were performed.

### AI-based segmentation

The body composition measurements are based on a convolutional neural network trained to segment a 3D CT image into SAT, muscle and others. The network architecture as well as the training pipeline is borrowed from Trägårdh et al. [18]. The network gives output scores ranging from 0 to 1 and normally a pixel would be assigned to the label with highest score, but to reduce noise, any scores not consistent with the HU thresholds above were first set to zero. Finally, the method from Trägårdh et al. [18] was used to find T11 and the hip bone, and the tissue segmentation was restricted to the CT slices in between.

### Statistical analysis

The Sørensen-Dice index was used to evaluate the accuracy of the AI-based method by calculating the overlap between the automated and the manual segmentations in the single CT slice at L3 level. The Wilcoxon signed rank test was used to evaluate the difference in reproducibility between the 2D areas at L3 level and the 3D volumes, both based on the AI-based method. The relative difference between the two AI-based volume measurements was calculated for the 74 pairs of CT studies. Linear regression models were applied for predicting 3D volumes of SAT and muscle from the corresponding 2D areas. The statistical analysis was carried out in R (version 4.0.3).

## Results

**Fig. 1:**
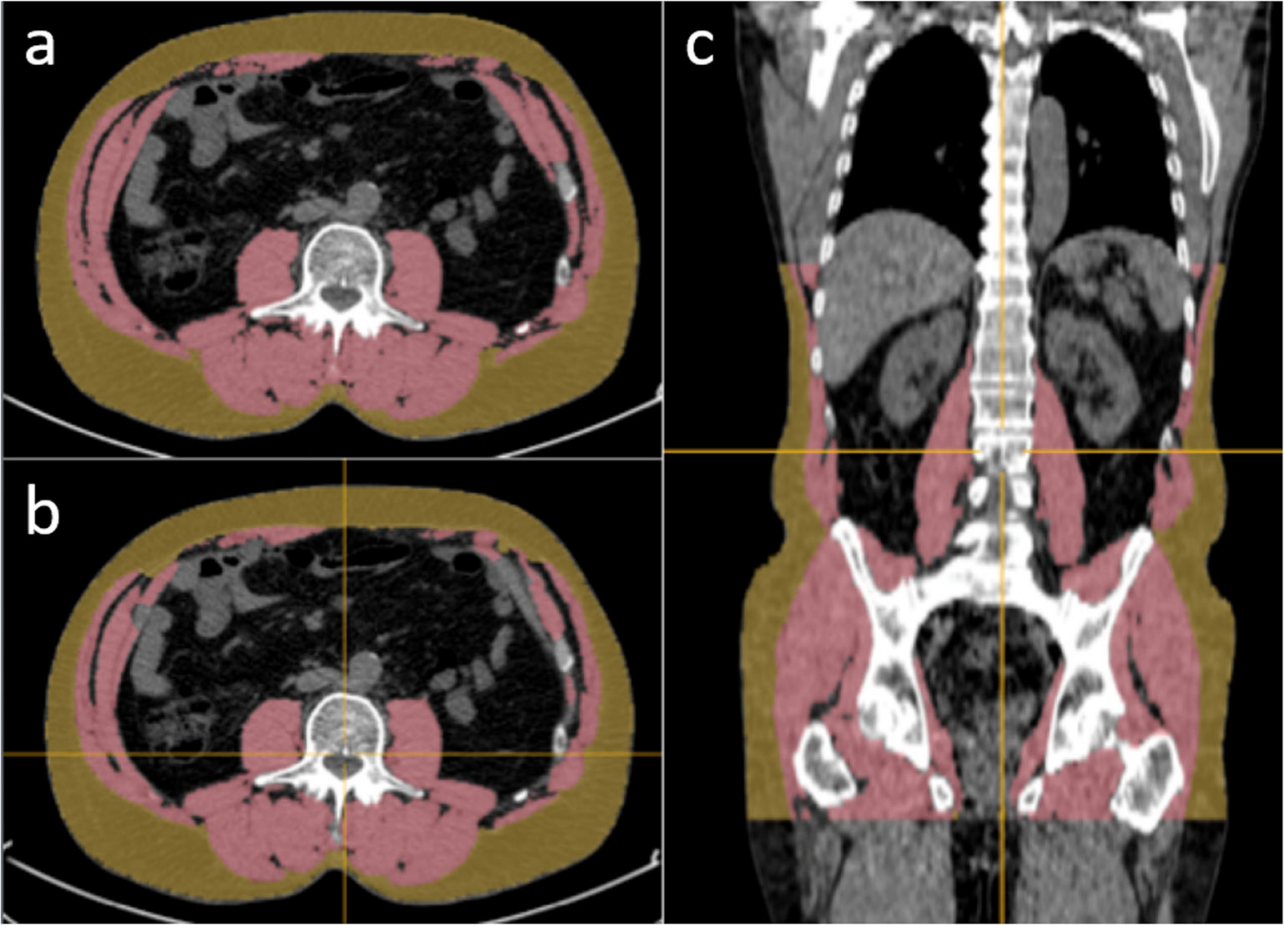
Manual and AI-based segmentations of SAT and muscle. Left: segmentation on a CT slice at L3 level: manual (**a**) and AI-based (**b**). Coronal slice showing the AI-based 3D segmentation from T11 to the hip bone (**c**). Measurements: manual areas, 186 cm^2^ (SAT) and 170 cm^2^ (muscle); AI-based areas, 184 cm^2^ (SAT) and 158 cm^2^ (muscle); AI-based volumes 6,832 cm^3^ (SAT) and 8,253 cm^3^ (muscle). *AI* Artificial intelligence, *SAT* Subcutaneous fat

The test group comprised 74 male patients with prostate cancer who had a mean age of 67 years (range 50–76) and a mean body weight of 86 kg (range 54–120). The median interval between the studies was 3 days. The AI-based method segmented SAT and muscle in 148 CT studies from those 74 patients from the cranial part of T11 to the caudal part of the hip bone. In 13/148 studies (9%), the AI-based method selected a vertebra above T11, and for these studies, the SAT and muscle segmentations of the slices above T11 were removed manually. No other manual corrections were applied. An example of manual and AI-based segmentations of SAT and muscle is shown in Fig. 1. The automated 3D segmentations covered on average 85 CT slices (range 77–98).

The average Sørensen-Dice index resulted to be 0.96 (range 0.86–0.99) for SAT and 0.94 (range 0.82–0.97) for muscle. The manual 2D areas, the AI-based 2D areas and the AI-based 3D volumes of SAT and muscle in the 74 patients are presented in Table 1, where also the differences for the same measurements calculated from the two CT scans from the same patient are shown. The AI-based 3D volumes showed a significantly better reproducibility, measured as lower relative inter-study differences, compared to the AI-based 2D areas. The relative inter-study differences for 3D volumes and 2D areas were 1.8% *versus* 5.0% for SAT (*p* < 0.001) and 1.9% *versus* 3.9% for muscle (*p* < 0.001).

**Table 1:** Subcutaneous adipose tissue and muscle areas and volumes calculated from manual and AI-based segmentations in the test group (74 patients and two studies each)

Segmentation	Area (cm^**2**^)/volume (cm^**3**^)	Difference (%)
**SAT**		
Manual 2D	191 (65–358)	5.5% (0.1–26.2)
AI-based 2D	190 (64–349)	5.0% (0.1-25.9)
AI-based 3D	7,386 (2,021–13,889)	1.8% (0.0–7.8)
**Muscle**		
Manual 2D	168 (90–229)	5.1% (0.0–19.7)
AI-based 2D	162 (92–217)	3.9% (0.0–15.9)
AI-based 3D	7,982 (5122–11,422)	1.9% (0.1–5.0)

Data are given as mean and ranges (minimum–maximum). *2D* Two-dimensional, *3D* Three-dimensional, *AI* Artificial intelligence, *SAT* Subcutaneous adipose tissue

**Fig. 2:**
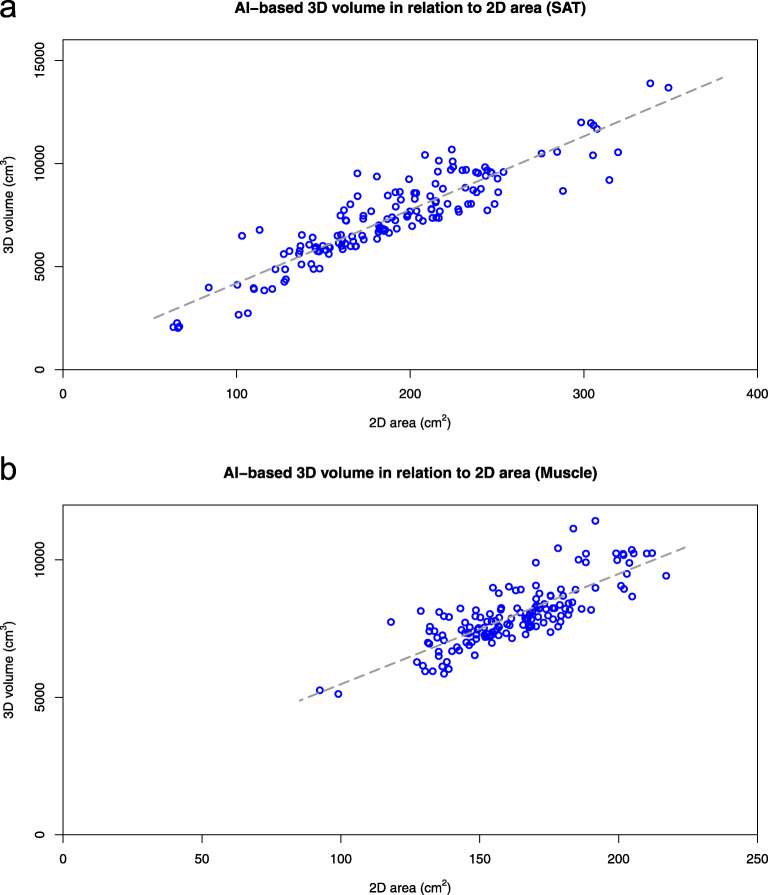
Relation between AI-based 3D volume and L3 slice 2D area for SAT (**a**) and muscle (**b**) for 148 computed tomography studies in 74 patients. *2D* Two-dimensional, *3D* Three-dimensional, *AI* Artificial intelligence, *SAT* Subcutaneous fat

The linear regression models used to predict the 3D volumes from the corresponding 2D areas, both segmented by the AI-based method, are presented in Fig. 2. For SAT, the linear model is

$$ y=35.63\ x+630.3 $$

with an *r*^2^ value of 0.83. For muscle, the estimated model is

$$ y=40.15\ x+1461 $$

with an *r*^2^ value of 0.64. If we consider a 95% prediction interval for an average subject´s 3D volume estimated from the 2D area, it had a size of ± 1.800 cm^3^ (24% of the average 3D volume) for SAT and ± 1.330 cm^3^ (17% of the average 3D volume) for muscle.

## Discussion

The AI-based method for automated segmentation of SAT and muscle showed a high accuracy when compared to manual segmentations on a CT slice at the L3 level, with a Sørensen-Dice index of 0.96 and 0.94, respectively. These results are comparable to previous published data [12–16]. The reproducibility was, as expected, significantly better for 3D volumes compared to 2D area measurements. The 95% confidence intervals for predicted 3D SAT and muscle volumes in an individual subject from the corresponding single CT slice areas resulted to be in the order of 25% and 17% of the average 3D volume of SAT and muscle, respectively. These volumes estimated from extrapolations from a single CT-slice area could lead to significant variations of actual volumes and hence affect clinical decisions and eventually prognosis. 3D volume variability obtained using extrapolations from single-CT areas is already a known phenomenon described almost 40 years ago [16]. Results obtained in our study are quite similar to those presented by Shen et al. and Greenfield et al. [11, 23].

The prediction error of volumes from single CT slice areas and the low reproducibility for these area measurements, obtained in our study, indicate that 3D volumes of SAT and muscle should be used instead of corresponding areas at L3 level in studies assessing the prognostic value of body composition.

The use of the CT performed in the PET/CT study for oncological patients to analyse body composition was an added benefit for the present study. In fact, the use of CT scans as part of PET/CT examination routinely performed in oncologic patients translates into a reduced radiation dose for patients and reduced costs for hospitals (medical equipment usage, health care personnel etc.) [24, 25].

The presented AI method is neither Conformité Européenne-marked in Europe nor Food and Drug Administration-cleared in the USA and therefore should considered a research tool, not ready for clinical use. It is, however, available for other researchers (www.recomia.org) who are interested in giving valuable input to what eventually can become a clinically available AI method.

Our study has limitations. First, we used manual segmentations of SAT and muscle in a single CT slice at L3 level to validate the AI-based method. A corresponding reference for the 3D volumes would have required manual segmentations of more than 12,000 CT slices (on average 85 slices each in 148 studies) and that was not possible due to time constraints. Based on the visual appearance of the 3D segmentations as in Fig. 1, our impression is that the AI-based method is accurate also for the volumetric measurements. Second, in 9% of the cases, a manual correction was needed due to difficulty to detect T11 by the AI-based tool. An alternative anatomical landmark than T11 could make the method more robust. Third, the VAT compartment was not included in the analysis. VAT analysis has been consistently been associated with poorer agreement with reference values [26–28] and carry a much more challenging analysis algorithm; due to those reasons, we decided not to include VAT in this first software version.

In conclusion, our AI-based tool for quantification of SAT and muscle volume from CT showed high accuracy and reproducibility. The tool is automated and provides a 3D analysis that is could be more clinically relevant than corresponding 2D methods for analysis of a single CT slice at the L3 level. Further studies are needed to assess the prognostic value of the AI tool, which is available for research purposes on reasonable request at www.recomia.org.

## Data Availability

The AI tool presented in this study is available for research purposes on reasonable request at www.recomia.org.

## Abbreviations

2D: Two-dimensional
3D: Three-dimensional
AI: Artificial intelligence
CT: Computed tomography
PET/CT: Positron emission tomography/computed tomography
SAT: Subcutaneous adipose tissue
VAT: Visceral adipose tissue

## References

[1] Brown JC, Cespedes Feliciano EM, Caan BJ (2018) The evolution of body composition in oncology-epidemiology, clinical trials, and the future of patient care: facts and numbers. J Cachexia Sarcopenia Muscle 9:1200–1208 doi: 10.1002%2Fjcsm.12379 10.1002/jcsm.12379 PMC6351674 30637983

[2] Kamarajah SK, Bundred J, Tan BH (2019). Body composition assessment and sarcopenia in patients with gastric cancer: a systematic review and metaanalysis. Gastric Cancer.

[3] Baracos VE, Reiman T, Mourtzakis M, Gioulbasanis I, Antoun S (2010). Body composition in patients with non− small cell lung cancer: a contemporary view of cancer cachexia with the use of computed tomography image analysis. Am J Clin Nutr.

[4] Hopkins JJ, Skubleny D, Bigam DL, Baracos VE, Eurich DT, Sawyer MB (2018). Barriers to the interpretation of body composition in colorectal cancer: a review of the methodological inconsistency and complexity of the CT-defined body habitus. Ann Surg Oncol.

[5] Trestini I, Carbognin L, Monteverdi S (2018). Clinical implication of changes in body composition and weight in patients with early-stage and metastatic breast cancer. Crit Rev Oncol Hematol.

[6] Peng YC, Wu CH, Tien YW, Lu TP, Wang YH, Chen BB (2020) Preoperative sarcopenia is associated with poor overall survival in pancreatic cancer patients following pancreaticoduodenectomy. Eur Radiol 10.1007/s00330-020-07294-7 10.1007/s00330-020-07294-7 32974690

[7] Lin TY, Peng CH, Hung SC, Tarng DC (2018). Body composition is associated with clinical outcomes in patients with non–dialysis-dependent chronic kidney disease. Kidney Int.

[8] Cruz-Jentoft AJ, Bahat G, Bauer J (2019). Sarcopenia: revised European consensus on definition and diagnosis. Age Ageing.

[9] Albano D, Messina C, Vitale J, Sconfienza LM (2020). Imaging of sarcopenia: old evidence and new insights. Eur Radiol.

[10] Marhold M, Topakian T, Unseld M (2020) Sarcopenia in cancer—a focus on elderly cancer patients. memo. 10.1007/s12254-020-00637-6

[11] Shen W, Punyanitya M, Wang Z (2004). Visceral adipose tissue: relations between single-slice areas and total volume. Am J Clin Nutr.

[12] Weston AD, Korfiatis P, Kline TL (2019). Automated abdominal segmentation of CT scans for body composition analysis using deep learning. Radiology.

[13] Lee H, Troschel FM, Tajmir S (2017). Pixel-level deep segmentation: artificial intelligence quantifies muscle on computed tomography for body morphometric analysis. J Digit Imaging.

[14] Paris MT, Tandon P, Heyland DK (2020). Automated body composition analysis of clinically acquired computed tomography scans using neural networks. Clin Nutr.

[15] Dabiri S, Popuri K, Feliciano EMC, Caan BJ, Baracos VE, Beg MF (2019). Muscle segmentation in axial computed tomography (CT) images at the lumbar (L3) and thoracic (T4) levels for body composition analysis. Comput Med Imaging Graph.

[16] Bridge CP, Rosenthal M, Wright B (2018). Fully-automated analysis of body composition from CT in cancer patients using convolutional neural networks. Radiology.

[17] Kjölhede H, Ahlgren G, Almquist H (2012). Combined 18F-fluorocholine and 18F-fluoride positron emission tomography/computed tomography imaging for staging of high-risk prostate cancer. BJU international.

[18] Trägårdh E, Borrelli P, Kaboteh R et al (2020) RECOMIA—a cloud-based platform for artificial intelligence research in nuclear medicine and radiology. EJNMMI physics 7:1–12. 10.1186/s40658-020-00316-9 10.1186/s40658-020-00316-9 PMC7403290 32754893

[19] Takahashi N, Sugimoto M, Psutka SP, Chen B, Moynagh MR, Carter RE (2017) Validation study of a new semi-automated software program for CT body composition analysis. Abdom Radiol (NY) 42:2369–2375. 10.1007/s00261-017-1123-6 10.1007/s00261-017-1123-6 28389787

[20] Tegels JJ, Van Vugt JL, Reisinger KW (2015). Sarcopenia is highly prevalent in patients undergoing surgery for gastric cancer but not associated with worse outcomes. J Surg Oncol.

[21] Feliciano EMC, Kroenke CH, Meyerhardt JA (2017). Association of systemic inflammation and sarcopenia with survival in nonmetastatic colorectal cancer: results from the C SCANS study. JAMA Oncol.

[22] Lee S, Janssen I, Ross R (2004) Interindividual variation in abdominal subcutaneous and visceral adipose tissue: influence of measurement site. J Appl Physiol (1985)97:948–954. 10.1152/japplphysiol.01200.2003 10.1152/japplphysiol.01200.2003 15121737

[23] Greenfield JR, Samaras K, Chisholm DJ, Campbell LV (2002). Regional intra-subject variability in abdominal adiposity limits usefulness of computed tomography. Obes. Res..

[24] Decazes P, Metivier D, Rouquette A, Talbot J-N, Kerrou K (2016). A method to improve the semiquantification of 18F-FDG uptake: reliability of the estimated lean body mass using the conventional, low-dose CT from PET/CT. J. Nucl. Med..

[25] Decazes P, Tonnelet D, Vera P, Gardin I (2019). Anthropometer3D: automatic multi-slice segmentation software for the measurement of anthropometric parameters from CT of PET/CT. J. Digit. Imaging..

[26] Kullberg J, Hedström A, Brandberg J (2017). Automated analysis of liver fat, muscle and adipose tissue distribution from CT suitable for large-scale studies. Sci. Rep..

[27] Kullberg J, Ahlström H, Johansson L, Frimmel H (2007) Automated and reproducible segmentation of visceral and subcutaneous adipose tissue from abdominal MRI. Int. Int J Obes 31:1806–1817. 10.1038/sj.ijo.0803671 10.1038/sj.ijo.0803671 17593903

[28] Makrogiannis S, Caturegli G, Davatzikos C, Ferrucci L (2013). Computer-aided assessment of regional abdominal fat with food residue removal in CT. Acad. Radiol..

